# The evaluation of the disease advancement in patients with mucopolysaccharidosis

**DOI:** 10.1186/1546-0096-12-S1-P153

**Published:** 2014-09-17

**Authors:** Violetta Opoka-Winiarska

**Affiliations:** 1 Department of Pediatric Pulmonology and Rheumatology, Medical University of Lublin, Lublin, Poland

## Introduction

One of the most important manifestations of mucopolysaccharidosis (MPS) type I, II and VI is a progressive disease of the osteoarticular system. The evaluation of the disease advancement is difficult due to the complexity of symptoms. The characteristic features are progressive limitation of joint mobility and joint pain. These symptoms affect the quality of patient life. A uniform scale has not been developed for these patients.

## Objectives

The aim of this study was to use the experience in the evaluation of disorders in rheumatic diseases (Juvenile Idiopathic Arthritis, JIA) in patients with MPS.

## Methods

6 patients with MPS VI were evaluated: 2 with advanced disease, 2 with moderate and 2 with slow progressing disease. The following parameters were selected for assessment: Physician global assessment of disease activity (PGA), Patient/parent global assessment of well-being (PGE), Functional ability (CHAQ), Number of joints with limited movement (LJC) and VAS pain – visual analogue scale for pain.

## Results

The evaluation results are shown in Table 1.

**Table 1:** 

Patient	Age (years)	PGA 0-10cm	PGE 0-10cm	CHAQ 0-3	LJC 0-71	VAS pain 0-10cm	Score (104)	Severity of disease
1	35	1.0	0	0.375	2	1.0	4.375	mild
	
2	21	2.0	0	1.125	2	0	5.125	↓
	
3	9	2.0	0	0	7	2.0	9	↓
	
4	16	2.0	0	0.125	8	0	10.125	↓
	
5	5	3.0	1,0	0	21	1.0	26	↓
	
6	11	3.0	0	0.125	37	3.5	43.625	advanced

## Conclusion

The parameters used in JIA may be applied for assessment of the MPS severity. With their implementation, the progression of the disease and the effect of the treatment can be assessed and compared.

## Disclosure of interest

None declared

